# The thermodynamic opportunities hypothesis: Metabolic temperature insensitivity across flatfish species

**DOI:** 10.1126/sciadv.adz0425

**Published:** 2026-02-25

**Authors:** Brad A. Seibel

**Affiliations:** College of Marine Science, University of South Florida, St. Petersburg, FL, USA.

## Abstract

This study challenges widely held metabolic theory, which suggests that whole-animal metabolic rates increase with temperature because of its universal effects on the kinetics of the underlying biochemical reactions. Here, we show that metabolic rates across flatfish species are largely invariant from poles to the equator, which points to an explanation for interspecific thermal sensitivity based on ecology and evolution rather than thermodynamic constraints. The explanation proposed here is that warm water provides a thermodynamic opportunity, not a mandate, for metabolic rate escalation when required for predator-prey interactions. Flatfish do not require metabolic escalation because of their reliance on camouflage that mitigates the greater predation intensity in tropical waters. These findings have strong implications for models attempting to diagnose the response of organisms to climate change and for macroecological patterns more generally.

## Teaser

Flatfish metabolism is temperature-insensitive, suggesting an evolutionary explanation rather than a thermodynamic constraint.

## INTRODUCTION

Ecological theory holds that a species’ metabolic rate, as a measure of resource uptake and allocation, constrains ecological processes across scales (*1*). Warming due to climate change is expected to elevate metabolic rates in ectotherms, potentially outpacing oxygen supply, thereby restricting growth and altering their biogeographical distributions (*2*–*4*). The intraspecific temperature sensitivity of metabolism in ectotherms is unexpectedly consistent and is well described by the Boltzmann-Arrhenius model, suggesting strict thermodynamic or kinetic underpinnings (*5*–*9*). Certainly, before acclimation or adaptation, an acute increase in temperature will exacerbate proton leak and diffusive ion loss, protein degradation, and other processes that contribute to elevated maintenance metabolism. The increase in metabolic rate upon exposure to high temperature has been observed both within and across species and is, thus, a central tenet of widely held (*1*) ecological theories.

**Fig. 1.**
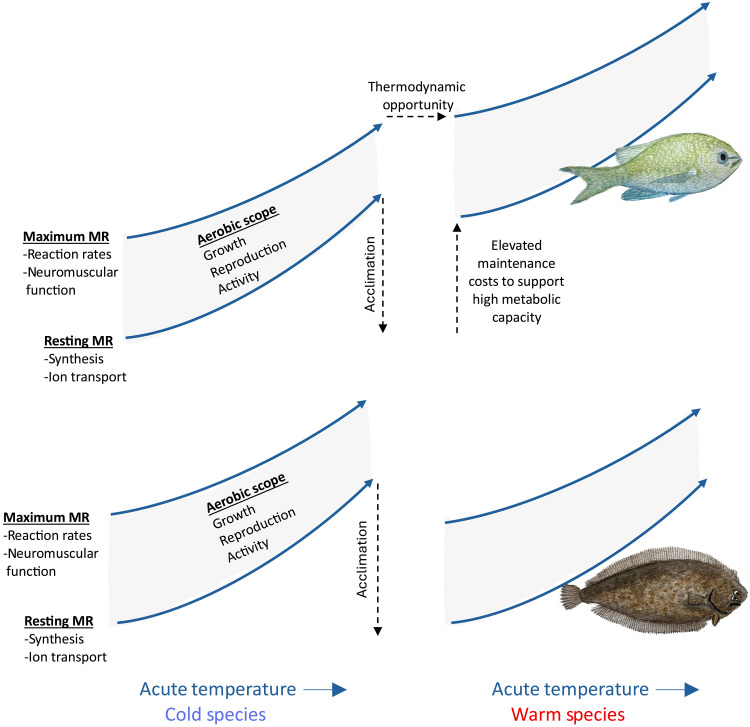
Schematic illustrating the thermodynamic opportunities hypothesis. The initial increase in RMRs in response to acute temperature change among ectotherms can be counteracted to some degree in the short term by adjustments to, for example, membrane and protein structure (acclimation, dashed arrows). In the absence of demand for high activity, acclimation would result in a similar range in metabolic rates (MRs) among ecologically similar species living across a latitudinal gradient (flatfish, bottom). For active species (top), escalating predator-prey interactions in warm waters will select for elevated activity and high active metabolic rates, above and beyond any acclimatory adjustments. The machinery supporting high active rates necessarily elevates maintenance metabolism as well. Thus, chronic exposure to warm waters leads to elevated rates among more active species despite acclimation. Artwork provided with permission from S. Emmons.

However, metabolism is tightly regulated and responds, via natural selection, to ecological pressures that are expected, on longer timescales and across species, to effectively decouple whole-animal metabolic rate from the kinetics of the underlying biochemical reactions (*10*–*16*). Thus, on longer timescales and across species, natural selection may adjust or compensate for those acute thermal effects on metabolic rate (Fig. 1). Compensation for such costs should reduce rates of maintenance metabolism and increase the aerobic scope among warm-adapted species (*17*–*20*). However, others report evidence of metabolic cold adaptation, an elevation in aerobic capacity among high-latitude species that compensates for the depressing effects of low temperature on mitochondrial adenosine 5′-triphosphate production (*15*, *21*–*25*). The net result in either case would be a flattening of the interspecific temperature-response curve, but the theoretical underpinnings are debated (*16*, *26*, *27*). The question remains: If physiology, ecology, and evolution are primary determinants of metabolic rate, and if acclimation is capable of compensating for thermodynamic effects, why are inter- and intraspecific temperature sensitivities of whole-animal metabolic rate consistently similar (*5*, *7*)?

Here, we hypothesize that increasing temperature has two distinct effects on metabolism. First, it elevates costs, such as increased ion loss, proton leak, or protein degradation, that primarily contribute to the standard, resting metabolic rate (RMR). Those costs can be, and often are, compensated through acclimation or acclimatization (*28*). Acclimation does not, however, appear to alter the thermal sensitivity across species in most interspecific studies to date. Thus, the cross-species thermal response curve usually resembles the acute, intraspecific response to temperature. We propose that this is because temperature also elevates metabolic potential, which, when realized, enhances species’ ability to acquire and use energy. This second effect of temperature provides an opportunity, rather than a mandate, for metabolic escalation. In that context, temperature has merely a permissive influence on the evolved metabolic rates across ectotherm species. Temperature acclimation and adaptation work on fundamentally different processes, responding to different selective pressures with the same underlying thermodynamic underpinnings. So, inter- and intraspecific temperature sensitivities may be similar, but they do not have to be.

High temperature permits higher absolute rates of biochemical reactions and physiological processes (e.g., muscle contraction and signal transduction) that support higher-order activities, such as locomotion. Higher activity must be supported by higher metabolic rates, regardless of temperature. Temperature, itself, does not provide a mechanistic drive for high metabolic rates or for the adoption of costly, high-activity lifestyles. It does, however, affect the fundamental ecology of resident organisms, necessitating temperature-specific performance that may reflect, or deviate from, the fundamental thermal sensitivity of chemical reactions (*29*, *30*). As a result, species that are engaged in active predator-prey interactions may compete in a temperature-mediated evolutionary arms race, experiencing positive selection for higher levels of performance at low latitudes [compare (*7*, *31*, *32*)]. While such selection would act on active, rather than resting, metabolic rates, active and resting rates are closely tied, driven by relaxed selection for energy conservation at rest in more active species (*20*, *33*–*35*). Because it is possible to have higher metabolic rates at high temperatures, many species do. However, in the absence of ecological demand for high rates, this thermodynamic opportunities hypothesis predicts that temperature will have little, if any, effect on metabolic rate across species. Benthic, sessile, or infaunal taxa have greater opportunities for refuge and cryptic camouflage or are otherwise removed from the demand for mobility for predator escape or prey capture. As such, they may be expected to have lower interspecific metabolic temperature sensitivities than mobile, pelagic taxa.

To test the thermodynamic opportunities hypothesis, we have compiled all available RMRs and related traits for flatfish (order Pleuronectiformes). Pleuronectiformes is a monophyletic group (*36*) of teleost fish that provides a unique comparative system. Flatfish are strongly laterally compressed, with both eyes residing on one side of the body. This unique morphological asymmetry facilitates an inactive existence, buried in the sediment and relying on camouflage with the seafloor to avoid predators and ambush prey. While some variability exists in behavior and predation strategies, the flatfish habit specifically requires prolonged inactivity in all species for which behavior has been described (*37*). Thus, we hypothesized that they may not avail themselves of the hypothesized thermodynamic opportunity for metabolic escalation in warm water. Note that the hypothesis applies as well if flatfish originated in the tropics, as recently suggested (*38*), and regardless of whether the flatfish’s inactive lifestyle is derived or ancestral. Metabolic rate is expected to diverge with predation pressure along a latitudinal temperature gradient, regardless of the directionality of that response.

We know of no other single monophyletic group of ectotherms that (i) are as speciose, (ii) occupy such a broad thermal range, (iii) are so ecologically and behaviorally similar, and (iv) are so widely studied with regard to metabolic rates. Of the 73 available studies, 15 studies were excluded for methodological reasons (see Materials and Methods, table S1, and fig. S1). The 30 remaining species represent about 5% of the extant Pleuronectiformes species. The dataset includes hundreds of individual metabolic rate measurements across four orders of magnitude in body size, 1000 m in depth, and 30°C in measurement temperature (table S2).

## RESULTS

**Fig. 2.**
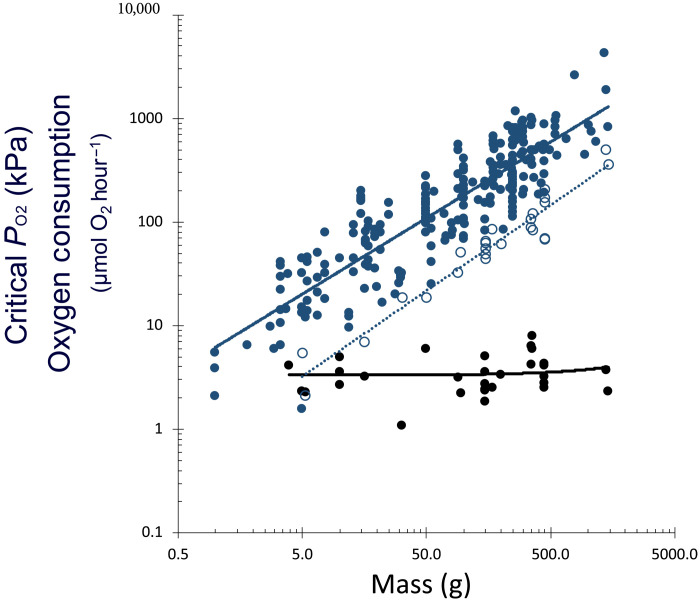
Scaling of metabolic traits with body mass in flatfish. Whole-animal RMR (blue circles) increases with a slope (*b* = 0.74 ± 0.048) indistinguishable from the mean intraspecific scaling coefficient (*b* = 0.73 ± 0.06). The critical oxygen partial pressure (black circles) does not change significantly with body mass. The oxygen supply capacity (open blue circles) scales with a slope (*b* = 0.82) statistically indistinguishable from that of RMRs.

Within each species, the whole-animal metabolic rate (*R*) increased with body mass (*M*) with a mean scaling coefficient, *b*, of 0.73 ± 0.06 (Eq. 1, *n* = 33), which was nearly identical to the scaling coefficient derived using all available data (i.e., including all individual measurements for all species; *b* = 0.74 ± 0.048; *n* = 246; Fig. 2). The 95% confidence intervals do not include values of 1 or 0.66. The scaling coefficient was unaffected by temperature across species.

$$R=R_{0}M^{b}$$ (1)

**Fig. 3.**
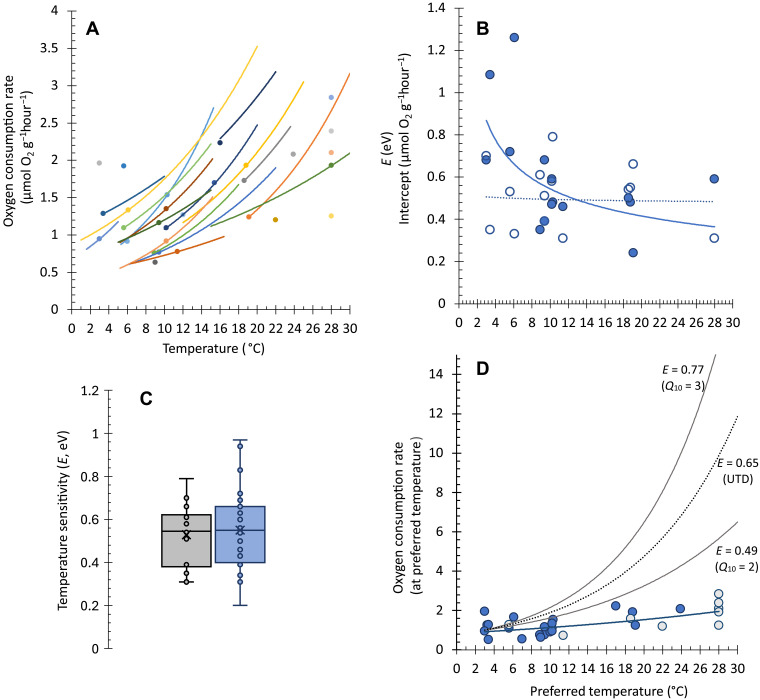
Temperature sensitivity of RMR in flatfish. (**A**) Acute exposure to rising temperature causes an increase in metabolic rate in each species, represented by different colors. The dots indicate the preferred habitat temperature for each species. Dots not associated with a line were for species measured at only one temperature. (**B**) The intercept (closed symbols) of each curve in (A) decreases with increasing temperature. The slopes (open symbols) are not temperature-dependent. (**C**) Temperature sensitivities of RMR (*E*) for each species (mean = 0.53 ± 0.15 eV; gray) and for each study (mean = 0.55 ± 0.18; some species were measured in more than one study; blue). (**D**) The RMR of each species at its preferred habitat temperature is relatively independent of temperature (*E* = 0.21 ± 0.11; *Q*_10_ = 1.30). Metabolic rates for the monophyletic superfamilies, Pleuronectoidea (blue) and Soleoidea (gray), are statistically indistinguishable. For comparison, more typical temperature relationships are also shown, including in parentheses the *Q*_10_ values of 2 and 3 and the universal temperature dependence [UTD; 0.65 eV; (*1*)].

Using this scaling coefficient, RMR was adjusted to a common body mass of 200 g (near the median mass for all measurements) for comparison across temperatures. At that common body mass, the metabolic rates of flatfish are low relative to commonly studied salmonid fish but are higher than deep-sea anglerfish (*35*). However, interspecific comparisons are complicated by the temperature relationships discussed here. During acute exposure to elevated temperature, the mass-normalized RMR of each species increased with temperature (*T*) as described by the Arrhenius relationship (Eq. 2 and Fig. 3A) with a coefficient (*E*) ranging from 0.31 to 0.79 (mean = 0.53 ± 0.15 eV; *n* = 16 species; Fig. 3, B and C, and Eq. 2, where *k* is Boltzmann’s constant). For species measured in multiple studies, measurements were combined to produce a single temperature coefficient [*E* (eV)] for each species (table S1) after first assuring that the acclimation temperatures were similar. These coefficients were calculated over temperature ranges of 4° up to 20°C (mean range = 10 ± 5°C). Temperature coefficients were derived from a minimum of three temperatures and from as many as 15. The mean temperature coefficient for individual studies was similar to the mean for species (0.55 ± 0.18; *n* = 27). The temperature coefficients were not significantly related to temperature (Fig. 3B).

$$e^{-E/\mathrm{kT}}$$ (2)

Using the species-specific temperature coefficients, we calculated the metabolic rate for each species at its mean preferred habitat temperature as reported by Fishbase (www.fishbase.org; table S1 and Fig. 3D). The habitat temperature preferences listed in Fishbase agree well with those reported independently by Cheung and Oyinlola (*39*).

In support of the thermodynamic opportunities hypothesis, the natural log of the mass-normalized RMR was relatively independent of temperature (1/*k*_B_*T*) across diverse flatfish species (Arrhenius plot, −0.21 ± 0.11*x* + 8.91, *R*^2^ = 0.35, *n* = 30, *P* = 0.001; Fig. 3D). The interspecific temperature sensitivity, derived from the slope of that relationship (*E* = 0.21; *Q*_10_ ~ 1.30), is well below that typically reported for marine ectotherms [*E* = 0.69, *Q*_10_ ~ 2.5; (*7*)] and the oft-cited universal temperature dependence [UTD; *E* = 0.65 eV; (*1*)]. This suggests that when inhabiting their preferred native habitat, all flatfish species have similarly low metabolic demands regardless of temperature.

## DISCUSSION

Flatfish metabolic rates have been studied for at least a century (*40*) and have been a target of several previous analyses of metabolic temperature sensitivity (*41*–*43*). These previous studies found no significant deviation from the supposed universal temperature dependence, perhaps due to low sample size or because they mixed intra- and interspecific analyses. In contrast, the present analysis finds that although the metabolic rate of each species increases with temperature, all flatfish species have similar metabolic rates when measured at their preferred habitat temperature.

The thermodynamic opportunities hypothesis explains this relative temperature invariance among flatfish as a result of compensated metabolic costs associated with acute temperature change and the absence of selection for metabolically expensive activity in sluggish flatfish. This hypothesis is conceptually akin to the geometric opportunities hypothesis regarding metabolic scaling (*44*), in which body size provides opportunities for energy savings rather than constraints on oxygen or nutrient uptake. Thermodynamic opportunities predict that selection for metabolic escalation is weaker in benthic and sessile taxa, a prediction supported by the present results and by a reportedly low temperature sensitivity across benthic fish (*45*, *46*) and sessile calcified invertebrates (*47*). However, it also explains the positive relationship between metabolism and temperature that has been observed in the vast majority of interspecific studies of marine ectotherms to date. We propose that there is a strong selective advantage for elevated metabolic rates to support increased activity for predator-prey interactions at a high temperature (*7*, *31*, *32*) and a high temperature provides a physiological opportunity to achieve that high activity. This hypothesis also allows for the existence of a wide range of metabolic rates at a common temperature as is observed between disparate species in any given habitat. Ecological demand for activity can select for enhanced oxidative metabolism even in extreme cold (*48*, *49*), while its absence permits low metabolic rates for some species occupying warm tropical waters. In general, however, species have realized the potential for high metabolic rates in warm waters leading to metabolic escalation, the magnitude of which approximates the thermodynamic effects on individual chemical reactions.

As pointed out by several earlier studies, adenosine 5′-triphosphate synthesis will not occur in the absence of increased energy demand. The demand that is driven purely by acute thermal effects is wasteful and is often counteracted or compensated on longer timescales (*13*, *26*, *27*). The question, then, is whether adapted metabolic rates also increase with temperature with a sensitivity that reflects the thermal sensitivity of the underlying biochemical reactions. We argue here that acute and chronic thermal sensitivities have different root causes and their magnitudes reflect different constraints and selective pressures. The distinction between a permissive environment or an environmental constraint is not merely semantic, even though, in the case of metabolic temperature sensitivity, the end result is often the same (i.e., elevated metabolic rate at high temperatures). The distinction is important because it influences where we may look, and how we might test, for variation from a typical temperature effect and, thus, how widespread we may find such variation to be.

Many recent models aim to predict the response of marine animals to climate change. Most of these models posit that an imbalance between environmental oxygen supply and metabolic oxygen demand constrains species’ fitness and biogeography. The predictions emerging from such models depend critically on the metabolic temperature sensitivity used. Models either assume a universal temperature dependence near *E* = 0.65 eV (*3*, *39*), use intraspecific (acute) temperature sensitivities derived from laboratory experiments (*2*, *4*, *50*), or infer thermal sensitivities from the biogeography of the species (*51*, *52*). None of these methods provides a parsimonious test of the mechanistic link between environmental oxygen and temperature and species’ distributions. Metabolic temperature sensitivity (or the lack there of) may be an effect, rather than a cause, of species’ biogeography (*29*, *53*). Because metabolic traits evolve to meet the ecological and environmental demands of the native habitat (*20*), the congruence of biogeographical range limits with physiological performance limits necessarily follows. Furthermore, in all of these models, there is an implicit assumption that metabolic rates will not be substantially altered via acclimation or adaptation to temperature on timescales relevant to anthropogenic climate change. The fact that flatfish show only a small effect of temperature across species suggests that they are quite adept at metabolic adjustment at least on evolutionary timescales. Cheung *et al.* (*54*) suggested that there may be standing genetic variability that would allow species to adapt evolutionarily to warming, while transgenerational (epigenetic) responses may also be possible for a few studied species. Many fish species appear to compensate over days or weeks for the acute effects of temperature on metabolic rates (*17*, *55*–*60*). Acclimation to different temperatures for even 1 month results in substantial adjustment of metabolic rate in several Pleuronectoidei flatfish (*61*–*63*). This is consistent with the finding of Slesinger *et al.* (*64*) that the habitat limits predicted from metabolic traits of summer flounder were not applicable across the entire latitudinal range occupied by this genetically homogeneous population. It is further consistent with recent isotopic estimates of field metabolic rate in the European plaice, which were “decoupled from seasonal variation in experienced temperature” (*65*).

Nonetheless, these metabolic models make bold predictions regarding loss of habitat (*51*), fisheries collapse (*39*), and growth potential (*41*) of flatfish. The present analysis challenges these predictions. The thermal sensitivity for California halibut, estimated by Franco *et al.* (*51*) from biogeographical data, was far outside the range of values found for any flatfish in the present study and for any other marine species to date (*66*). As a result, they predicted a 50% loss in metabolically available habitat by the end of the 21st century. Given that the temperature sensitivity of this species has not been directly measured, perhaps caution is warranted in evaluating this study’s prediction.

**Fig. 4.**
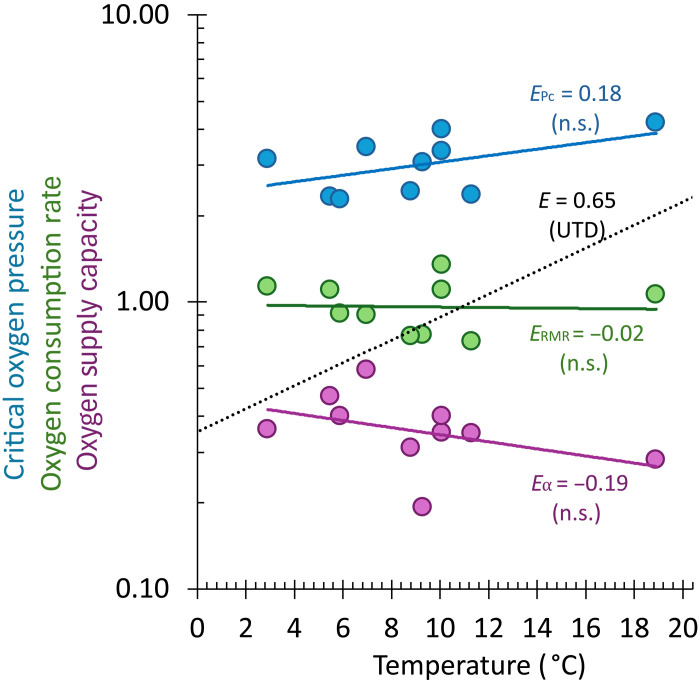
Metabolic traits are insensitive to temperature. The RMR (green), oxygen supply capacity (α; purple), and critical oxygen partial pressure (*P*_c_; blue) were available and could be normalized to the preferred habitat temperature for a small set of species (*n* = 10). None of the three metrics change significantly with temperature over the range available. n.s., not significant.

Pauly (*41*) also reported a more typical metabolic temperature sensitivity in flatfish (*Q*_10_ ~ 2) and concluded that higher metabolic rates at higher temperatures in tropical flatfish reduce the ultimate size at which oxygen limits their growth. This was posited to be the reason that flatfish tend to reach a larger size at a high latitude. This hypothesis is refuted by the present analysis. Furthermore, the physiological capacity to supply oxygen (α) far exceeds the resting metabolic demand at all sizes and temperatures (*20*). Across species, the mass-scaling coefficients for α are statistically indistinguishable from those for RMR (*b*_α_ = 0.82). Within the smaller subset of species for which α could be ascertained, RMR, the critical oxygen pressure (*P*_c_), and α were all independent of temperature (*P* > 0.05, *n* = 11 species; Fig. 4). Thus, physiological oxygen limitation cannot explain the reduced growth and body size in tropical flatfish species.

The present analysis reveals the relative thermal insensitivity of metabolic rates across flatfish species, contradicting the vast majority of studies on ectotherms, including previous analyses on flatfish (*3*, *39*, *41*, *50*, *51*). The finding counters the notion that thermodynamic effects on biochemical kinetics mandate increased metabolism. Instead, we suggest that increasing temperature provides an opportunity, not a mandate, for metabolic escalation. That opportunity is under intense selection to combat increasing predation intensity in warm water [compare (*30*)]. Given their sluggish lifestyle, flatfish instead conserve energy in their benthic habitat and do not elevate metabolism with increasing temperature. Understanding the nature of thermal effects on metabolism is of critical importance for predicting the effects of ocean warming and deoxygenation on the biogeography of marine animals.

## MATERIALS AND METHODS

For all available species of postmetamorphic Pleuronectoidei, we endeavored to extract from the literature every RMR measurement and any associated metabolic traits (e.g., *P*_cRMR_; table S1). We also extracted or calculated intraspecific mass and temperature scaling coefficients where possible (table S2). We conducted extensive literature searches using a broad range of search terms related to metabolism and to flatfish, including individual taxonomic names. We also searched the reference list of each relevant paper to identify additional studies missed in the original search. For each extracted measurement, we recorded (table S1) available data on fish mass, measurement temperature, acclimation temperature and duration, capture location and temperature, measurement oxygen partial pressures, respirometry trial duration, feeding history, and animal condition (resting, active, etc.).

In total, we analyzed data from 73 studies encompassing 34 species in six families. Of those studies, 15 did not meet the minimum requirements we set for inclusion (tables S1 and S2). Specifically, they provided measurements made on postsurgical specimens or provided fewer than 3 hours of time in the respirometry chamber inclusive of acclimation and measurement (table S1). Several studies noted anecdotally that 3 hours was sufficient time to recover from handling stress in flatfish, although one study found a much more gradual recovery (*61*). For four species, all available studies were among the 15 excluded. Thus, the final count of included species is 30. All studies included at least 12-hour laboratory (temperature) acclimation and fasting before trial initiation. One study (including two species) could not be eliminated on methodological grounds but nonetheless produced data that were about five times higher than all other studies (*67*). Those outliers are included in the table but not in any analysis. RMR was reported for all species. Additional metabolic traits, including maximum metabolic rate, critical oxygen partial pressures (*P*_c_), metabolic scaling, and temperature coefficients, were available for a subset of species.

Acclimation history and respirometry methods, as well as the reporting of methods, differed considerably between studies, which, in some instances, confounded interpretation. The included studies used flow-through, intermittent-flow, static, or swim-tunnel respirometry with and without sand or other substrate in the measurement chamber. The number of individual fish enclosed in the respirometry chambers also varied between studies. The numerous methodological variants precluded independent statistical analysis of the effects of any single variant on the reported rates. However, recent feeding, activity, and surgical procedures did appear to result in elevated metabolic rates. Although not readily testable, the methodology did not appear to vary with temperature in any way that would influence the conclusions of this meta-analysis with the possible exception of acclimation time. Acclimation within the chamber of 2 to 3 hours appeared sufficient to result in a resting status in most studies. Warmer temperatures often resulted in shorter chamber acclimation periods and shorter trials. Thus, while chamber acclimation may have influenced the results here, it would have exaggerated the effect of temperature, resulting in artificially elevated temperature sensitivities in contrast to the relatively low values reported here. Laboratory acclimation times (before trial) varied substantially but not systematically across temperatures, latitude, or body size. While the variability in methodology between studies could produce variable data that may obscure temperature trends, the data are constrained within a fairly tight range (±~40%). Most studies did not provide sufficient information about capture locations and local hydrography to address the effect of capture conditions or seasonal acclimatization.

The monophyly of the suborder Pleuronectoidei (order Carangiformes), which contains all but one family of flatfish, is broadly supported (*38*, *68*, *69*). However, the relationships within this suborder are not fully resolved. Two distinct superfamilies, Soleoidea and Pleuronectoidea, contained sufficient thermal breadth and a sufficient sample size to test independently for the thermal sensitivity of metabolic rate. While the representative Soleoidea is, on average, from warmer environments than Pleuronectoidea, each group spanned 20°C in preferred habitat temperature, and the thermal sensitivities of each group were low (*E* < 0.24 eV) and statistically indistinguishable from each other (Fig. 3D).

## Supplementary Materials

### The PDF file includes:

Fig. S1 Legends for tables S1 and S2 References
