# Supplementary material for: The thermodynamic opportunities hypothesis: Metabolic temperature insensitivity across flatfish species
Source: Sci Adv. 2026 Feb 25;12(9):eadz0425. doi: 10.1126/sciadv.adz0425 (PMC12935043; doi:10.1126/sciadv.adz0425)
Supplement: Supplementary file 1 — Fig. S1 Legends for tables S1 and S2 References [file sciadv.adz0425_sm.pdf]

Science Advances
AAAS

Supplementary Materials for

The thermodynamic opportunities hypothesis: Metabolic temperature insensitivity across flatfish species

Brad A. Seibel

Corresponding author: Brad A. Seibel, seibel@usf.edu

Sci. Adv. **12**, eadz0425 (2026)
DOI: 10.1126/sciadv.adz0425

The PDF file includes:

Fig. S1
Legends for tables S1 and S2
References

Other Supplementary Material for this manuscript includes the following:

Tables S1 and S2

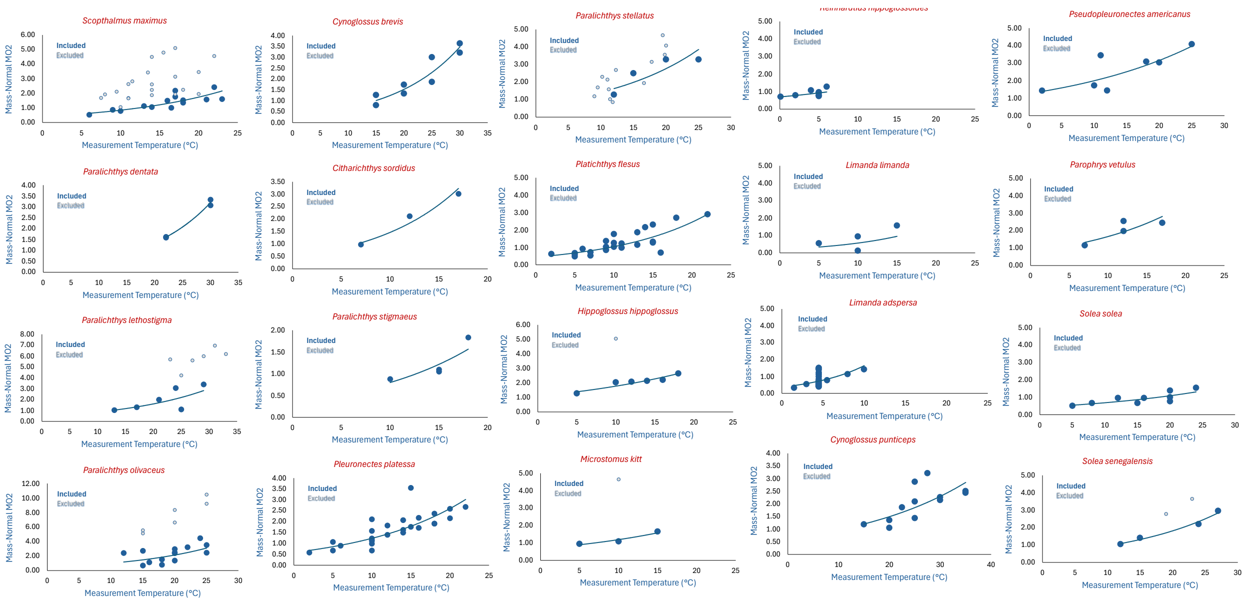

Figure S1. Relationships between resting metabolic rate (mass normalized) with measurement temperature by species. Each graph combines separate studies on the same species. Some studies used methods that are incompatible with resting metabolic rate (e.g. feeding, surgical procedures) and were exluded from analyses. Those data are indicated with gray symbols. The equations describing the curves in this figure are provided in Table S1. The raw data are included in Table S2

Auxiliary Files

Table S1 (Excel File). Summary of studies used to create temperature relationships.

Table S2 (Excel file). Raw metabolic rate data for flatfish species.

REFERENCES

1. J. H. Brown, J. F. Gillooly, A. P. Allen, V. M. Savage, G. B. West, Toward a metabolic theory of ecology. *Ecology* **85**, 1771–1789 (2004).

2. C. Deutsch, A. Ferrel, B. Seibel, H.-O. Pörtner, R. B. Huey, Climate change tightens a metabolic constraint on marine habitats. *Science* **348**, 1132–1135 (2015).

3. W. W. L. Cheung, J. L. Sarmiento, J. Dunne, T. L. Frölicher, V. W. Y. Lam, M. L. Deng Palomares, R. Watson, D. Pauly, Shrinking of fishes exacerbates impacts of global ocean changes on marine ecosystems. *Nat. Clim. Change* **3**, 254–258 (2013).

4. J. G. Rubalcaba, W. C. E. P. Verberk, A. J. Hendriks, B. Saris, H. A. Woods, Oxygen limitation may affect the temperature and size dependence of metabolism in aquatic ectotherms. *Proc. Natl. Acad. Sci. U.S.A.* **117**, 31963–31968 (2020).

5. J. F. Gillooly, A. P. Allen, V. M. Savage, E. L. Charnov, G. B. West, J. H. Brown, Response to Clarke and Fraser: Effects of temperature on metabolic rate. *Funct. Ecol.* **20**, 400–404 (2006).

6. J. P. Delong, J. P. Gilbert, T. M. Luhring, G. Bachman, B. Reed, A. Neyer, K. L. Montooth, The combined effects of reactant kinetics and enzyme stability explain the temperature dependence of metabolic rates. *Ecol. Evol.* **7**, 3940–3950 (2017).

7. A. I. Dell, S. Pawar, V. M. Savage, Temperature dependence of trophic interactions are driven by asymmetry of species responses and foraging strategy. *J. Anim. Ecol.* **83**, 70–84 (2014).

8. P. M. Schulte, The effects of temperature on aerobic metabolism: Towards a mechanistic understanding of the responses of ectotherms to a changing environment. *J. Exp. Biol.* **218**, 1856–1866 (2015).

9. M. J. Angilletta, R. B. Huey, M. R. Frazier, Thermodynamic effects on organismal performance: Is hotter better? *Physiol. Biochem. Zool.* **83**, 197–206 (2010).

10. W. Wieser, in *Effects of Temperature on Ectothermic Organisms: Ecological Implications and Mechanisms of Compensation*, W. Wieser, Ed. (Springer-Verlag, New York Heidelberg Berlin, 1973), pp. 1–24.

11. A. Clarke, Is there a universal temperature dependence of metabolism? *Funct. Ecol.* **18**, 252–256 (2004).

12. A. Clarke, K. P. P. Frazer, Why does metabolism scale with temperature? *Funct. Ecol.* **18**, 243–251 (2004).

13. P. W. Hochachka, in *Biochemistry and Molecular Biology of Fishes*, P. W. Hochachka, T. Mommsen, Eds. (Elsevier Science Publishers, 1991), vol. 1.

14. G. Chaui-Berlinck, C. A. Navas, H. A. Monteiro, J. E. P. W. Bicudo, Temperature effects on a whole metabolic reaction cannot be inferred from its components. *Proc. R. Soc. B* **271**, 1415–1419 (2004).

15. C. R. White, L. A. Alton, P. B. Frappell, Metabolic cold adaptation in fishes occurs at the level of whole animal, mitochondria and enzyme. *Proc. R. Soc. B* **279**, 1740–1747 (2012).

16. E. Uliano, A. Chaurasia, L. Berna, C. Agnisola, G. D'Onofrio, Metabolic rate and genomic GC. What we can learn from teleost fish. *Mar. Genomics* **3**, 29–34 (2010).

17. F. Jutfelt, Metabolic adaptation to warm water in fish. *Funct. Ecol.* **34**, 1138–1141 (2020).

18. F. Jutfelt, T. Norin, E. R. Asheim, L. E. Rowsey, A. H. Andreassen, R. Morgan, T. D. Clark, B. Speers-Roesch, Aerobic scope protection reduces ectotherm growth under warming. *Funct. Ecol.* **35**, 1397–1407 (2021).

19. E. Sandblom, T. D. Clark, A. Grans, A. Ekstrom, J. Brijs, L. Frerik Sunström, A. Odelstrom, A. Adill, T. Aho, F. Jutfelt, Physiological constraints to climate warming in fish follow principles of plastic floors and concrete ceilings. *Nat. Commun.* **7**, 11447 (2016).

20. B. A. Seibel, C. Deutsch, Oxygen supply capacity in animals evolves to meet maximum demand at the current oxygen partial pressure regardless of size or temperature. *J. Exp. Biol.* **223**, jeb210492 (2020).

21. D. E. Wohlschlag, Metabolism of an Antarctic fish and the phenomenon of cold adaptation. *Ecology* **41**, 287–292 (1960).

22. P. F. Scholander, W. Flagg, V. Walters, L. Irving, Climatic adaptation in Arctic and tropical poikilotherms. *Physiol. Zool.* **26**, 67–92 (1953).

23. R. G. M. Wells, Respiration of Antarctic fish from McMurdo sound. *Comp. Biochem. Physiol. A Comp. Physiol.* **88**, 417–424 (1987).

24. A. Addo-Bediako, S. L. Chown, K. J. Gaston, Metabolic cold adaptation in insects: A large-scale perspective. *Funct. Ecol.* **16**, 332–338 (2002).

25. J. C. Rosenthal, B. A. Seibel, A. Dymowska, F. Bezanilla, Trade-off between aerobic capacity and locomotory activity in an Antarctic pteropod. *Proc. Natl. Acad. Sci. U.S.A.* **106**, 6192–6196 (2009).

26. A. Clarke, What is cold adaptation and how should we measure it? *Integr. Comp. Biol.* **31**, 81–92 (1991).

27. A. Clarke, Seasonal acclimatization and latitudinal compensation in metabolism: Do they exist? *Funct. Ecol.* **7**, 139–149 (1993).

28. L. Gerber, K. A. Clow, A. K. Gamperl, Acclimation to warm temperatures has important implications for mitochondrial function in Atlantic salmon (*Salmo salar*). *J. Exp. Biol.* **224**, jeb236257 (2021).

29. B. A. Seibel, M. A. Birk, Unique metabolic temperature sensitivity creates a cold-water habitat barrier in diel vertical migrators. *Nat. Clim. Change* **12**, 1052–1058 (2022).

30. G. V. Ashton, A. L. Freestone, J. E. Duffy, M. E. Torchin, Predator control of marine communities increases with temperature across 115 degrees latitude. *Science* **376**, 1215–1219 (2022).

31. L. E. Culler, M. A. McPeek, M. P. Ayres, Predation risk shapes thermal physiology of a predaceous damselfly. *Oecologia* **176**, 653–660 (2014).

32. L. A. Twardochileb, T. C. Treakle, P. L. Zarnetske, Foraging strategy mediates ectotherm predator-prey responses to climate warming. *Ecology* **101**, e03146 (2020).

33. S. K. Auer, S. S. Killen, E. L. Rezende, Resting vs. active: A meta-analysis of the intra- and inter-specific associations between minimum, sustained, and maximum metabolic rates in vertebrates. *Funct. Ecol.* **31**, 1728–1738 (2017).

34. K. Reinhold, Energetically costly behaviour and the evolution of resting metabolic rate in insects. *Funct. Ecol.* **13**, 217–224 (1999).

35. B. A. Seibel, J. C. Drazen, The rate of metabolism in marine animals: Environmental constraints, ecological demands and energetic opportunities. *Philos. Trans. R. Soc. Lond. B Biol. Sci.* **362**, 2061–2078 (2007).

36. R. R. Betancur, G. Orti, Molecular evidence for the monophyly of flatfishes (Carangimorpharia: Pleuronectiformes). *Mol. Phylogenet. Evol.* **73**, 18–22 (2014).

37. R. N. Gibson, A. W. Stoner, C. H. Ryer, in *Flatfishes: Biology and Exploitation*, R. N. Gibson, R. D. M. Nash, A. J. Geffen, H. W. van der Veer, Eds. (John Wiley & Sons, ed. 2, 2015), chapter 12.

38. M. A. Campbell, B. Chanet, J.-N. Chen, M.-Y. Lee, W.-J. Chen, Origins and relationships of the Pleuronectoidei: Molecular and morphological analysis of living and fossil taxa. *Zool. Scr.* **48**, 640–656 (2018).

39. W. W. L. Cheung, M. A. Oyinlola, Vulnerability of flatfish and their fisheries to climate change. *J. Sea Res.* **140**, 1–10 (2018).

40. F. M. Baldwin, Comparative rates of oxygen consumption in marine forms. *Proc. Iowa Acad. Sci.* **30**, 173–180 (1923).

41. D. Pauly, A framework for latitudinal comparisons of flatfish recruitment. *Neth. J. Sea Res.* **32**, 107–118 (1994).

42. A. Clarke, N. M. Johnston, Scaling of metabolic rate with body mass and temperature in teleost fish. *J. Animal Ecol.* **68**, 893–905 (1999).

43. L. I. Karamushko, Metabolic adaptation of fish at high latitudes. *Dokl. Biol. Sci.* **379**, 359–361 (2001).

44. J. J. Childress, G. N. Somero, Metabolic scaling: A new perspective based on scaling of glycolytic enzyme activities. *Am. Zool.* **30**, 161–173 (1990).

45. I. A. Johnston, A. Clark, P. Ward, Temperature and metabolic rate in sedentary fish from the Antarctic, North Sea, and Indo-West Pacific Ocean. *Mar. Biol.* **109**, 191–195 (1991).

46. Y. Watanabe, N. L. Payne, Thermal sensitivity of metabolic rate mirrors biogeographic differences between teleosts and elasmobranchs. *Nat. Commun.* **14**, 2054 (2023).

47. S.-A. Watson, S. A. Morley, A. E. Bates, M. S. Clark, R. W. Day, M. Lamare, S. M. Martin, P. C. Southgate, K. Siang Tan, P. A. Tyler, L. S. Peck, Low global sensitivity of metabolic rate to temperature in calcified marine invertebrates. *Oecologia* **174**, 45–54 (2014).

48. A. Dymowska, T. Manfreddi, J. C. Rosenthal, B. A. Seibel, Muscle ultrastructure and mitochondrial morphometrics in polar and temperate pteropods (Gymnosomata: Gastropoda). *J. Exp. Biol.* **215**, 3370–3378 (2012).

49. E. L. Crockett, B. D. Sidell, Some pathways of energy metabolism are cold adapted in Antarctic fishes. *Physiol. Zool.* **63**, 472–488 (1990).

50. V. Freitas, S. A. L. M. Kooijman, H. W. van der Veer, Latitudinal trends in habitat quality of shallow-water flatfish nurseries. *Mar. Ecol. Prog. Ser.* **471**, 203–214 (2012).

51. A. C. Franco, H. Kim, H. Frenzel, C. Deutsch, D. Ianson, U. Rachid Sumaila, Impact of warming and deoxygenation on the habitat distribution of Pacific halibut in the Northeast Pacific. *Fish. Oceanogr.* **31**, 601–614 (2022).

52. E. H. Howard, J. L. Penn, H. Frenzel, B. A. Seibel, D. Bianchi, L. Renault, F. Kessouri, M. A. Sutula, J. C. McWilliams, C. Deutsch, Climate driven aerobic habitat loss in the California Current System. *Sci. Adv.* **6**, eaay3188 (2020).

53. B. A. Seibel, in *Encyclopedia of Fish Physiology*, S. L. Alderman, T. E. Gillis, Eds. (Elsevier, Academic Press, 2024), vol. 3, pp. 548–560.

54. W. W. L. Cheung, G. Reygondeau, T. L. Frolicher, Large benefits to marine fisheries of meeting the 1.5°C global warming target. *Science* **80**, 1591–1594 (2016).

55. A. D. Zambie, K. L. Ackerly, B. J. Negrete, A. J. Esbaugh, Warming-induced "plastic floors" improve hypoxia vulnerability, not aerobic scope, in red drum (*Sciaenops ocellatus*). *Sci. Total. Environ.* **922**, 171057 (2024).

56. F. Seebacher, C. R. White, C. E. Franklin, Physiological plasticity increases resilience of ectothermic animals to climate change. *Nat. Clim. Change* **6**, 61–66 (2014).

57. E. Sandblom, A. Gräns, M. Axelsson, H. Seth, Temperature acclimation rate of aerobic scope and feeding metabolism in fishes: Implications in a thermally extreme future. *Proc. R. Soc. B* **281**, 20141490 (2014).

58. N. Pilakouta, S. S. Killen, B. K. Kristjansson, S. Skulason, J. Lindstrom, N. B. Metcalfe, K. J. Parsons, Multigenerational exposure to elevated temperatures leads to a reduction in standard metabolic rate in the wild. *Funct. Ecol.* **34**, 1205–1214 (2020).

59. J. C. Havird, J. L. Neuwald, A. A. Shah, A. Mauro, C. A. Marshall, C. K. Ghalambor, Distinguishing between active plasticity due to thermal acclimation and passive plasticity due to Q_{10} effects: Why methodology matters. *Funct. Ecol.* **34**, 1015–1028 (2020).

60. H. E. Wooten, J. R. Morrongiello, T. Schmitt, A. Audzijonyte, Smaller adult fish size in warmer water is not explained by elevated metabolism. *Ecology* **25**, 1177–1188 (2022).

61. G. G. Duthie, D. F. Houlihan, The effect of single step and fluctuating temperature changes on the oxygen consumption of flounders, *Platichthys flesus* (L.): Lack of temperature adaptation. *J. Fish Biol.* **21**, 215–226 (1982).

62. M. Jobling, A study of some factors affecting rates of oxygen consumption of plaice, *Pleuronectes platessa* L. *J. Fish Biol.* **20**, 501–516 (1982).

63. A. Gräns, F. Jutfelt, E. Sandblom, E. Jonsson, K. Wiklander, H. Seth, C. Olsson, S. Dupont, O. Ortega-Martinez, I. Einarsdottir, B. Thrandur Bjornsson, K. Sundell, M. Axelsson, Aerobic scope fails to explain the detrimental effects on growth resulting from warming and elevated CO_2 in Atlantic halibut. *J. Exp. Biol.* **217**, 711–717 (2014).

64. E. Slesinger, H. duPontavice, B. A. Seibel, V. S. Saba, J. Kohut, G. K. Saba, Climate-induced reduction in metabolically suitable habitat for U.S. Northeast shelf marine species. *PLOS Clim.* **3**, e0000357 (2024).

65. J. Jones, E. Hunter, C. Trueman, Seasonal and sex-based variations in energy use in wild plaice: Differences in energetic costs of spawning and feeding behaviours outweigh effects of temperature on field metabolism. *Fish. Res.* **2889**, 107476 (2025).

66. C. Deutsch, J. L. Penn, B. A. Seibel, Metabolic trait diversity shapes marine biogeography. *Nature* **585**, 557–562 (2020).

67. J. Davenport, E. Kjorsvik, T. Haug, Appetite, gut transit, oxygen uptake and nitrogen excretion in captive Atlantic halibut, *Hippoglossus hippoglossus* L., and lemon sole, *Microstomus kitt* (Walbaum). *Aquaculture* **90**, 267–277 (1990).

68. W. Shi, S. Chen, X. Kong, L. Si, L. Gong, Y. Zhang, H. Yu, Flatfish monophyly refereed by the relationship of *Psettodes* in Carangimorphariae. *BMC Genomics* **19**, 400 (2018).

69. Z. Lü, L. Gong, Y. Ren, Y. Chen, Z. Wang, L. Liu, H. Li, X. Chen, Z. Li, H. Luo, H. Jiang, Y. Zeng, Y. Wang, K. Wang, C. Zhang, H. Jiang, W. Wan, Y. Qin, J. Zhang, L. Zhu, W. Shi, S. He, B. Mao, W. Wang, X. Kong, Y. Li, Large-scale sequencing of flatfish genomes provides

insights into the polyphyletic origin of their specialized body plan. *Nat. Genet.* **53**, 742–751 (2021).

70. M. P. Armstrong, A comparative study of the ecology of smooth flounder, Pleuronectes putnami, and winter flounder, Pleuronectes americanus, from great bay estuary, New Hampshire, thesis, University of New Hampshire (1995).

71. H. Bergsson, H. R. Andersen, M. B. S. Svendsen, P. J. Hansen, J. F. Steffensen, Respiratory physiology of European Plaice (*Pleuronectes platessa*) exposed to *Prymnesium parvum*. *Fishes* **4**, 32 (2019).

72. J. A. G. Brown, A. Jones, A. J. Matty, Oxygen metabolism of farmed turbot (*Scophthalmus maximus*) I. The influence of fish size and water temperature on metabolic rate. *Aquaculture* **36**, 273–281 (1984).

73. C. Burel, J. Person-Le Ruyet, F. Gaumet, A. Le Roux, A. Severe, G. Boeuf, Effects of temperature on growth and metabolism in juvenile turbot. *J. Fish Biol.* **49**, 678–692 (1996).

74. K. M. Caposella, R. W. Brill, M. C. Fabrizio, P. G. Bushnell, Metabolic and cardiorespiratory responses of summer flounder *Paralichthys dentatus* to hypoxia at two temperatures. *J. Fish Biol.* **81**, 1043–1058 (2012).

75. J. J. Cech Jr., D. M. Rowell, J. S. Glasgow, Cardiovascular responses of the winter flounder, *Pseudopleuronectes americanus*, to hypoxia. *Comp. Biochem. Physiol. A* **57**, 123–125 (1977).

76. J. W. F. Chu, K. S. P. Gale, Ecophysiological limits to aerobic metabolism in hypoxia determine epibenthic distributions and energy sequestration in the northeast Pacific ocean. *Limnol. Oceanogr.* **62**, 59–74 (2017).

77. J. C. Cornett, S. L. Hamilton, C. A. Logan, Physiological sensitivities to hypoxia differ between co-occurring juvenile flatfishes. *J. Exp. Mar. Biol. Ecol.* **578**, 152033 (2024).

78. A. Z. Dalla Valle, R. Rivas-Diaz, G. Claireaux, Opercular differential pressure as a predictor of metabolic oxygen demand in the starry flounder. *J. Fish Biol.* **63**, 1578–1588 (2003).

79. F. M. Del Toro-Silva, J. M. Miller, J. C. Taylor, T. A. Ellis, Influence of oxygen and temperature on growth and metabolic performance of *Paralichthys lethostigma* (Pleuronectiformes: Paralichthyidae). *J. Exp. Mar. Biol. Ecol.* **358**, 113–123 (2008).

80. C. Dietz, K. T. Stiller, M. Griese, C. Schulz, A. Susenbeth, Influence of salinity on energy metabolism in juvenile turbot, *Psetta maxima* (L.). *Aquac. Nutr.* **19**, 135–150 (2013).

81. A. Dupont-Prinet, M. Vagner, D. Chabot, C. Audet, Impact of hypoxia on the metabolism of Greenland halibut (*Reinharditius hippoglossoides*). *Can. J. Fish. Aquat. Sci.* **70**, 461–469 (2013).

82. G. G. Duthie, The respiratory metabolism of temperature-adapted flatfish at rest and during swimming activity and the use of anaerobic metabolism at moderate swimming speeds. *J. Exp. Biol.* **97**, 359–373 (1982).

83. R. R. C. Edwards, D. M. Finlayson, J. H. Steele, The ecology of 0-group Plaice and common dabs in Loch Ewe. II. Experimental studies of metabolism. *J. Exp. Mar. Biol. Ecol.* **3**, 1–17 (1969).

84. R. R. C. Edwards, J. H. S. Blaxter, U. K. Gopalan, C. V. Mathew, D. M. Finlayson, Feeding, metabolism, and growth of tropical flatfish. *J. Exp. Mar. Biol. Ecol.* **6**, 279–300 (1971).

85. J. D. Findlay, Physiological Responses of Bluethroat Wrasse, Notolabrus tetricus, Horseshoe Leatherjacket, Meuschenia hippocrepis and Greenback Flounder, Rhombosolea tapirina, to Low Temperature Transport, thesis, University of Tasmania (2005).

86. M. Fonds, R. Cronie, A. D. Vethaak, P. Van Der Puyl, Metabolism, food consumption and growth of plaice (*Pleuronectes platessa*) and flounder (*Platichthys flesus*) in relation to fish size and temperature. *Neth. J. Sea Res.* **29**, 127–143 (1992).

87. C. P. Hickman, The osmoregulatory role of the thyroid gland in the starry flounder, *Platichthys stellatus*. *Can J. Zool.* **37**, 998–1060 (1959).

88. Y. Hishida, H. Katch, T. Oda, A. Ishimatsu, Comparison of physiological responses to exposure to *Chattonella marina* in Yellowtail, Red Sea Bream and Japanese Flounder. *Fish. Sci.* **64**, 875–881 (1998).

89. J. G. Hoff, Lethal oxygen concentration for three marine fish species. *J. Water Pollut. Control Fed.* **39**, 267–277 (1967).

90. H. Honda, Displacement behavior of Japanese flounder, *Paralichthys olivaceus*, estimated by the difference of oxygen consumption rate. *Nippon Suisan Gakkaishi* **54**, 1259 (1988).

91. A. K. Imsland, A. Fokvord, S. O. Stefansson, Growth, oxygen consumption and activity of juvenile turbot (*Scopthalmus maximus L.*) reared under different temperatures and photoperiods. *Neth. J. Sea Res.* **34**, 149–159 (1995).

92. A. Imsland, A. Foss, B. Sveinsbo, T. M. Jonassen, S. O. Stefansson, Comparisons of RNA/DNA ratios, growth, and metabolism in different populations of juvenile turbot, *Scopthalmus maximus*, reared at four temperatures. *J. World Aquac. Soc.* **32**, 1–10 (2001).

93. M.-H. Jeong, Y. S. Kim, B. H. Min, Y. J. Chang, Effects of water temperature on oxygen consumption in starry flounder, *Platichthys stellatus*, reared in seawater and freshwater. *Korean J. Environ. Biol.* **27**, 285–291 (2009).

94. Y. Jia, J. Wang, Y. Gao, B. Huang, Hypoxia tolerance, hematological, and biochemical response in juvenile turbot (*Scopthalmus maximus. L*). *Aquaculture* **535**, 736380 (2021).

95. L. Jones, The thermal physiology of yellowbelly flounder (Rhombosolea leporina) in a coastal, New Zealand Lake, thesis, University of Cantebury (2021).

96. J. B. Jorgensen, T. Mustafa, The effect of hypoxia on carbohydrate metabolism in flounder (*Platichthys flesus L.*)—I. Utilization of glycogen and accumulation of glycolytic end products in various tissues. *Comp. Biochem. Physiol. B* **67**, 243–248 (1980).

97. A. Kerstens, J. P. Lomholt, K. Johansen, The ventilation, extraction and uptake of oxygen in undisturbed flounders, *Platichthys flesus*: Responses to hypoxia acclimation. *J. Exp. Biol.* **83**, 169–179 (1979).

98. I.-N. Kim, Y.-J. Chang, J.-Y. Kwon, The patterns of oxygen consumption in six species of marine fish. *J. Korean Fish. Soc.* **28**, 373–381 (1995).

99. J. Lee, Postprandial ammonia excretion and oxygen consumption rates in olive flounder Paralichthys olivaceus fed two different feed types according to water temperature change. *Fish. Aquat. Sci.* **18**, 373–378 (2015).

100. H. Liu, Y. Sakurai, H. Munehara, K. Shimazaki, Diel rhythms of oxygen consumption and activity level of juvenile flounder *Paralichthys olivaceus*. *Fish. Sci.* **63**, 655–658 (1997).

101. P. F. MacIsaac, G. P. Goff, D. J. Speare, Comparison of routine oxygen consumption rates of three species of pleuronectids at three temperatures. *J. Appl. Ichthyol.* **13**, 171–176 (1997).

102. R. Mallekh, J. P. La-gardere, Effect of temperature and dissolved oxygen concentration on the metabolic rate of the turbot and the relationship between metabolic scope and feeding demand. *J. Fish Biol.* **60**, 1105–1115 (2002).

103. V. K. Maxime, K. Pichavant, G. Boeuf, G. Nonnotte, Effects of hypoxia on respiratory physiology of turbot, *Scopthalmus maximus*. *Fish Physiol. Biochem.* **22**, 51–59 (2000).

104. G. E. Merino, R. H. Piedrahita, D. E. Conklin, Routine oxygen consumption rates of California halibut (*Paralichthys californicus*) juveniles under farm-like conditions. *Aquac. Eng.* **41**, 166–175 (2009).

105. M. L. Moser, S. W. Ross, K. J. Sulak, Metabolic responses to hypoxia of *Lycenchelys verrillii* (wolf eelpout) and *Glyptocephalus cynoglossus* (witch flounder): Sedentary bottom fishes of the Hatteras/Virginia middle slope. *Mar. Ecol. Prog. Ser.* **144**, 57–61 (1996).

106. S.-Y. Oh, Y.-S. Jang, H.-S. Park, Y. U. Choi, C. K. Kim, The influence of water temperature and body weight on metabolic rate of Olive flounder *Paralichthys olivaceus*. *Ocean Polar Res.* **34**, 93–99 (2012).

107. J. O. Onukwufor, C. M. Wood, The osmorespiratory compromise in marine flatfish: Differential effects of temperature, salinity, and hypoxia on diffusive water flux and oxygen

consumption of English sole (*Parophyrs vetulus*) and Pacific sanddab (*Citharichthys sordidus*). *Mar. Biol.* **169**, 1–15 (2022).

108. A. J. Paul, J. M. Paul, R. L. Smith, Rates of oxygen consumption of yellowfin sole (*Limanda aspera* (Pallas)) relative to body size, food intake and temperature. *J. Cons. Int. Explor. Mer.* **47**, 205–207 (1990).

109. K. Pichavant, J. Person-Le-Ruyet, N. Le Bayon, A. Severe, A. Le Roux, L. Quemener, V. Maxime, G. Nonnotte, G. Boeuf, Effects of hypoxia on growth and metabolism of juvenile turbot. *Aquaculture* **188**, 103–114 (2000).

110. I. G. Priede, F. G. T. Holliday, The use of a new tilting tunnel respirometer to investigate some aspects of metabolism and swimming activity of the plaice (*Pleuronectes platessa L.*). *J. Exp. Biol.* **85**, 295–309 (1980).

111. E. J. Rupia, S. A. Binning, D. G. Roche, W. Lu, Fight-flight or freeze-hide? Personality and metabolic phenotype mediate physiological defence responses in flatfish. *J. Anim. Ecol.* **85**, 927–937 (2016).

112. A. Ruth, M. B. Sondergaard Svendsen, R. Nygaard, E. A. F. Christensen, P. G. Bushnell, J. F. Steffensen, Physiological effects of temperature on Greenland halibut *Reinhardtius hippoglossoides* shows high vulnerability of Arctic stenotherms to global warming. *J. Fish Biol.* **103**, 675–683 (2023).

113. G. D. Schweiterman, D. P. Crear, B. N. Anderson, D. R. Lavoie, J. A. Sulikowski, P. G. Bushnell, R. W. Brill, Combined effects of acute temperature change and elevated pCO_2 on the metabolic rates and hypoxia tolerances of clearnose skate (*Rostaraja eglanteria*), summer flounder (*Paralichthys dentatus*), and thorny skate (*Amblyraja radiata*). *Biology* **8**, 56 (2019).

114. J. F. Steffensen, J. P. Lomholt, J. L. Johansen, Gill ventilation and O_2 extraction during graded hypoxia in two ecologically distinct species of flatfish, the flounder (*Platichthys flesus*) and the plaice (*Pleuronectes platessa*). *Environ. Biol. Fish.* **7**, 157–163 (1982).

115. J. C. Taylor, J. M. Miller, Physiological performance of juvenile southern flounder, *Paralichthys lethostigma* (Jordan and Gilbert, 1884), in chronic and episodic hypoxia. *J. Exp. Mar. Biol. Ecol.* **258**, 195–214 (2001).

116. X. Tian, J. Fang, S. Dong, Effects of starvation and recovery on the growth, metabolism and energy budget of juvenile tongue sole (*Cynoglossus semilaevis*). *Aquaculture* **310**, 122–129 (2010).

117. L. Tito de Morais, Growth and respiration of two pleuronectiform juveniles from a western Mediterranean bay. *J. Fish Biol.* **27**, 459–468 (1985).

118. C. C. van Maaren, J. Kita, H. V. Daniels, in *Proceedings of the Twenty-Eighth US-Japan Natural Resources Aquaculture Panel*, C. C. T. Tamaru, C. S. Tamaru, J. P. McVey, K. Ikuta, Eds. (University of Hawai'i Sea Grant College Program, 2000).

119. C. Vinagre, L. Narciso, M. Pimentel, H. N. Cabral, M. J. Costa, R. Rosa, Contrasting impacts of climate change across seasons: Effects on flatfish cohorts. *Reg. Environ. Change* **13**, 853–859 (2013).

120. R. A. Voyer, G. E. Morrison, Factors affecting respiration rates of winter flounder (*Pseudopleuronectes americanus*). *J. Fish. Board Can.* **28**, 1907–1911 (1971).

121. U. Waller, Factors influencing routine oxygen consumption in turbot, *Scopthalmus maximus*. *J. Appl. Ichthyol.* **8**, 62–71 (1992).

122. C. M. Wood, B. McMahon, D. G. McDonald, Respiratory gas exchange in the resting starry flounder, *Platichthys stellatus*: A comparison with other teleosts. *J. Exp. Biol.* **78**, 167–179 (1979).

123. F. Gaumet, G. Boeuf, A. Severe, A. Le Roux, N. Mayer-Gostan, Effects of salinity on the ionic balance and growth of juvenile turbot. *J. Fish Biol.* **47**, 865–876 (1995).
